# The utility of the basophil activation test in the diagnosis of immediate amoxicillin or amoxicillin-clavulanate hypersensitivity in children and adults

**DOI:** 10.1186/s13052-017-0360-1

**Published:** 2017-04-21

**Authors:** Simona Barni, Francesca Mori, Claudia Valleriani, Giusi Mangone, Sergio Testi, Francesca Saretta, Lucrezia Sarti, Neri Pucci, Maurizio de Martino, Chiara Azzari, Elio Novembre

**Affiliations:** 1 Allergy Unit, A. Meyer Children’s Hospital, University of Florence, Viale Pieraccini, 24, 50134 Florence, Italy; 2 Departments of Paediatrics, A. Meyer’s Children Hospital, Viale Pieraccini, 24, 50139 Florence, Italy; 3 Allergy and Clinical Immunology Unit, San Giovanni di Dio’s Hospital, Florence, Italy; 4 Departments of Paediatrics, A.A.S 2 Bassa Friulana Palmanova’s Hospital, Udine, USA; 5 Department of Health Sciences, A. Meyer Children’s Hospital, University of Florence, Florence, Italy

**Keywords:** Adults, Amoxicillin, Basophil activation test, Children, Clavulanate acid

## Abstract

**Background:**

The basophil activation test (BAT), has been proposed as a possible assay for the diagnosis of immediate-type allergy to beta-lactams (BLs).

The aim of this study was to assess the utility of BAT in the diagnosis of amoxicillin (AMX) or AMX-clavulanate (AMX-C) IgE-mediated hypersensitivity in children and adults.

**Material and methods:**

Eighteen children and 21 adults, with clinical history of immediate reactions to AMX or AMX-C, were referred to Anna Meyer Children’s Hospital and San Giovanni di Dio Hospital, respectively. They underwent in vivo tests (skin prick test and intradermal test). Moreover, BAT with AMX or AMX-C was performed within 6 months from the reaction.

**Results:**

In the pediatric group, the concordance between the skin tests (ST) and BAT results was 83.3%. Upon comparing the symptom grades and ST results to the BAT results, we found that the reaction severity and ST positivity did not correlate with BAT results in children.

In the adult group, the concordance between the ST and BAT results was 61.9%. Upon comparing patients with severe reactions and patients with mild reactions in terms of BAT results, we found a BAT sensitivity of 38.5% and a specificity of 100%. When comparing the symptom grades to the BAT results, we found that no patients with mild symptoms had a positive BAT result, whereas 38.5% of patients with severe symptoms had a positive BAT result.

**Conclusions:**

BAT does not seem to be a useful tool to increase the sensitivity of an allergy work-up to diagnose immediate hypersensitivity to AMX or AMX-C.

## Dear Editor,

Beta-lactams (BLs) are the antibiotics that most frequently provoke hypersensitivity reactions, which have a prevalence rate of 5–10% in children and adults [1].

According to the European Network for Drug Allergy (ENDA), the diagnosis of immediate amoxicillin hypersensitivity should be based on the patient’s clinical history, skin test (ST) (skin prick test [SPT] and intradermal [ID] test) results, or the determination of the specific IgE to the beta-lactams and drug provocation test (OPT) results [2].

An in vitro test called the basophil activation test (BAT) has been proposed as a possible functional assay for the diagnosis of allergy to BLs. Upon challenge with specific allergens that cross-link membrane-bound IgE antibodies, basophils upregulated the expression of different activation markers, such as CD63 and CD203c. The BAT involves the detection of these immune-phenotypic alterations on a single-cell basis by multicolour flow cytometry using specific monoclonal antibodies [3].

The aim of this study was to assess the utility of the BAT in the diagnosis of amoxicillin (AMX) or AMX-clavulanate (AMX-C) IgE-mediated hypersensitivity in children and adults.

Charts from consecutive patients from January 2013 to January 2016 were reviewed. Data from patients referred to the Allergy Unit of Anna Meyer Children’s Hospital and San Giovanni di Dio Hospital for immediate reactions to AMX or AMX-C who underwent in vivo and in vitro tests, including BAT to AMX, AMX-C and C, within 6 months of the reaction were analysed.

Reactions to AMX or AMX-C were classified as immediate if they occurred within one hour after antibiotic intake in the first 24–36 h of treatment [2]. The generalized hypersensitivity reactions were graded as mild [reaction limited to skin tissue (urticaria, erythema and angioedema)]; moderate (features suggesting respiratory, cardiovascular or gastrointestinal involvement) or severe (hypoxia, hypotension or neurologic involvement) (4). Anaphylaxis was defined as “multiple-organ hypersensitivity characterized by the presence of significant gastrointestinal, respiratory, or cardiovascular involvement in addition to skin features” [4]. Anaphylaxis was considered to be a severe hypersensitivity reaction.

All parents of child patients or the adult patients themselves were asked to sign a written informed consent form before starting the allergy work-up. All patients had been off antihistamines or oral corticosteroids for 10 days before drug evaluation.

STs with the culprit drug were performed according to ENDA recommendations [5]. The only exception to this protocol was that we did not test the major penicillin determinant of benzylpenicilloyl conjugated to poly-L-lysine (PPL) or the mixture of minor determinants (MDM). Moreover, in cases with a positive ID to AMX-C, AMX was also tested (20 mg/ml).

Specific IgE to AMX, ampicillin, penicillin G and V and total IgE were measured in children with suspected immediate reactions (Immunocap-FEIA, Uppsala, Sweden). The positive cut-off was >0.10 KU/l.

For adult patients, the detection of specific IgE was not performed in cases involving positive ST results.

The BAT to AMX or AMX-C was carried out according to the protocol presented in Sanz et al [6].

Patients with negative ST and in vitro test results underwent an oral provocation test (OPT) to AMX or AMX-C [1/10-2/10-7/10 of the therapeutic dose (50 mg/kg/day in 2 doses) administered every 30 min] following current guidelines until the therapeutic cumulative dose was reached or until a reaction occurred [7]. In one case with borderline values of specific IgE to AMX (0.18 KU/l), a negative ST result and mild symptoms, OPT was also performed. The OPT was considered to be positive if any objective skin, respiratory and/or cardiovascular, neurologic, or gastrointestinal symptoms were recorded. Patients were observed for 2 h after the last drug intake if they had a negative outcome and 2 h after the resolution of symptoms in case of any reaction.

All statistical analyses were performed using SPSS (SPSS Inc. Chicago, IL, USA) version 16.0.

From January 2013 to January 2016, 18 children with a suspected past clinical history of reactions to AMX or AMX-C were included if they fit the inclusion criteria. In particular, they had to have had the BAT within 6 months of the reaction or positive ST results.

In the same period, 21 adults with a suspected past clinical history of immediate reaction to AMX or AMX-C who were referred to the San Giovanni di Dio Allergy Unit were enrolled.

Thirty-nine patients were studied, including 18 children (average age: 7.1 years; age range: 2 years-18 years; sex: 10 males and 8 females) and 21 adults (average age: 57 years; age range: 31 years-89 years; sex: 8 males and 13 females).

Their clinical and immunological features are summarized in Tables 1 and 2.

**Table 1:** Clinical and immunologic results in the children population

patient	1	2	3	4	5	6	7	8	9	10	11	12	13	14	15	16	17	18
sex	M	M	M	M	M	F	F	F	M	F	F	F	F	F	M	M	M	M
Age (years)	12	14	4	2	3	9	14	3	3	6	3	2	5	2	18	5	9	15
drug	A	AC	AC	AC	AC	AC	AC	AC	AC	AC	AC	AC	AC	AC	A	AC	AC	AC
Grading symptoms	Mi	S	Mi	Mo	Mi	Mi	Mi	Mi	Mi	Mi	Mi	Mi	Mi	Mi	Mi	Mi	Mi	Mi
SPT	neg	6/20	neg	neg	neg	neg	neg	neg	neg	neg	neg	neg	neg	neg	3/10	neg	neg	neg
ID	6/8	np	neg	neg	neg	neg	neg	neg	neg	neg	neg	neg	neg	neg	neg	neg	neg	neg
Specific IgE (KU/l)	neg	Amx 0,46	neg	neg	neg	neg	neg	neg	neg	neg	neg	neg	neg	neg	Amx 0,25	neg	neg	Amx 0,18
Total IgE (KU/l)	1951	2055	2	82	373	82	23	6	5	13	11	34	292	383	127	61	198	846
BAT	neg	neg	neg	neg	neg	neg	neg	neg	neg	neg	neg	neg	neg	neg	neg	neg	neg	neg
OPT	np	np	neg	np	neg	pos	pos	neg	neg	neg	neg	neg	neg	np	np	neg	neg	pos

*A* amoxicillin, *AC* amoxicillin + clavulanic acid, *BAT* basophil activation test, *F* female, *ID* intradermal test *M* male, *Mi* mild symptoms, *Mo* moderate symptoms, *neg* negative, *np*: not performed, *OPT* oral provocation test, *S* severe symptoms, *SPT* skin prick test

**Table 2:** Clinical and immunologic results in the adult population

patient	1	2	3	4	5	6	7	8	9	10	11	12	13	14	15	16	17	18	19	20	21
sex	F	F	F	F	F	F	F	F	F	M	M	M	M	M	M	F	F	M	M	M	M
Age (years)	79	65	57	31	66	65	53	69	69	60	52	27	48	48	73	31	58	73	73	73	73
drug	A	AC	AC	AC	AC	AC	AC	AC	AC	A	AC	AC	AC	AC	A	AC	AC	A	A	A	A
Grading symptoms	S*	S	Mi	S	S	Mi	Mi	S	S	S	S	Mi	Mi	S	S	Mi	S	S	S	S	S
SPT	pos	neg	neg	neg	neg	neg	neg	neg	neg	neg	neg	neg	neg	pos	neg	neg	neg	neg	neg	neg	neg
ID	np	pos	pos	neg	pos	neg	pos	pos	pos	pos	pos	pos	pos	np	pos	neg	neg	pos	pos	pos	pos
SpecificIgE (KU/l)	np	np	np	np	np	neg	np	np	np	np	np	np	np	np	np	Np	Amx 0,18	np	np	np	np
BAT	pos	pos	neg	neg	pos	neg	neg	neg	pos	neg	pos	neg	neg	neg	neg	neg	neg	neg	neg	neg	neg
OPT	np	np	np	neg	np	pos	np	np	np	np	np	np	np	np	np	neg	np	np	neg	neg	neg

*A* amoxicillin, *AC* amoxicillin + clavulanic acid, *BAT* basophil activation test, *F* female, *ID* intradermal test *M* male, *Mi* mild symptoms, *Mo* moderate symptoms, *neg* negative, *np* not performed, *S* severe symptoms, *SPT* skin prick test, * Skin prick test induced anaphylaxis

In the paediatric population, the culprit drug was AMX-C in sixteen out of eighteen patients (88.8%), and the route of administration was “per os”. Two children reacted to AMX. Eleven patients (61.1%) had already taken and tolerated the culprit drug prior to clinical manifestation of symptoms.

Urticaria was the most common symptom, occurring in 100% of patients. Eight patients (44.4%) had angioedema, two (11%) patients had dyspnoea and one patient (5%) reported vomiting.

The symptom grading was mild for sixteen patients (88.8%) and moderate for one patient (5.5%). Only one patient (5%) had severe symptoms.

The average latency time between drug intake and symptom occurrence was 32 min (DS ± 24).

Three patients had a positive ST result. Two patients had a positive SPT result at 20 mg/ml (one patient to AMX-C and the other one to AMX). The third patient had a positive AMX ID (20 mg/ml) result at immediate reading.

The test for specific IgE to AMX was positive in three out of eighteen patients. Two of those patients also had a positive ST result. The third patient, who was also provoked, had a positive OPT.

No one had a positive BAT result for AMX, AMX-C and C alone. Fifteen patients (83.3%) were both ST- and BAT-negative. Among those with either a positive or a negative ST result, three patients (16.6%) had a positive ST result and a negative BAT result (two patients with mild symptoms and one with moderate symptoms).

Consequently, concordance between the ST and BAT results was 83.3% (fifteen patients were both ST- and BAT-negative).

The average latency time between the reactions and BAT determination was 4 months (DS ± 2).

Upon comparing the symptom grades and ST results to the BAT results, we found that the reaction severity and ST positivity did not correlate with BAT results in children.

Thirteen out of eighteen patients underwent OPT, all with AMX-C. Five patients were not provoked (two patients refused, three patients had a positive ST result). Ten out of thirteen patients had a negative OPT result. All three patients with a positive OPT result reacted within one hour from the last drug intake. Two children had negative results on both the ST and test for specific IgE to AMX. One patient had a positive but weak result on the test for specific IgE to AMX.

In the adult population, the culprit drug was AMX-C in 18 patients (85.7%) and AMX in 3 patients (14.3%). The route of administration was “per os”.

All patients had already taken and tolerated the culprit drug before their clinical symptoms manifested.

Ten patients (47.6%) had erythema, nine patients (42.8%) had loss of consciousness, seven patients had itching (33.3%) without urticaria, six patients (28.5%) had urticaria, three patients (14.3%) had angioedema, three patients (14.3%) had dyspnoea, two patients (13.3%) had confusion, one patient (4.7%) had throat tightness, one patient (4.7%) had cyanosis, one patient (4.7%) had vomiting, and one patient (4.7%) had sweating. Most patients had more than one symptom. There were 45 clinical symptoms in total.

The symptoms were graded as mild for 8 patients (38.1%) and severe for 13 patients (61.9%).

The average latency time between culprit drug intake and the manifestation of clinical symptoms was 13 min and 30 s (DS ± 17).

ST results were positive in thirteen patients (61.9%). One patient had SPT-induced anaphylaxis with AMX (at 20 mg/ml concentration). One patient had a positive SPT result with AMX-C (at 20 mg/ml concentration). Eleven patients had a positive ID result with AMX-C (at 20 mg/ml concentration) and AMX (at 20 mg/ml concentration) at immediate reading.

The specific IgE was measured in three of the eight ST-negative patients. Only one had a positive specific IgE to AMX.

Five patients (23.8%) were both AMX-ST- and BAT-positive (all with severe symptoms). Eight patients had positive ST and negative BAT results (38.1%). Eight patients (38.1%) were both ST- and BAT- negative (one had a positive specific IgE to AMX). No patient had a C-positive BAT result.

Six patients underwent OPT with AMX-C, with only one having a positive result.

Consequently, the concordance between the ST and BAT results was 61.9% (five patients were both ST- and BAT-positive, eight patients were both ST- and BAT-negative).

In the adult population, upon comparing patients with severe reactions (13 out of 21) and patients with mild reactions (8 out of 21) in terms of BAT results, we found a BAT sensitivity of 38.5% and a specificity of 100%.

The average elapsed time between symptom appearance and the BAT was 6 months. We performed the BAT on four patients, even though the elapsed time was longer than six months because it was within six months of a positive ST result.

When comparing the symptom grades to the BAT results, we found that no patients with mild symptoms had a positive BAT result, whereas 5 out of 13 patients (38.5%) with severe symptoms had a positive BAT result.

A summary of children and adult’s results are showed in Table 3.

**Table 3:** Summary of children and adult’s results

	Children Number of patients/total; (%)	Adults Number of patients/total; (%)	p
Grading symptoms:			
• Mild	16/18; (88.8)	8/21; (38.10)	0.020
• Moderate	1/18; (5.5)	0/21; (0)	0.317
• Severe	1/18; (5.5)	13/21; (61.90)	0.001
AMX positive ST	3/18; (16.6)	13/18;(72.2)	0.002
AMX positive BAT	0/18; (0)	5/21; (23.8)	0.025
AMX or AMX-C positive OPT	3/13; (23.07)	1/6; (16.6)	0.157

*AMX* amoxicillin, *C* clavulanate acid, *BAT* basophil activation test, *OPT* oral provocation test, *ST* skin test

This is the first study that assessed the utility of the BAT in the diagnosis of IgE-mediated hypersensitivity to AMX or AMX-C in children with a history of immediate reactions.

Moreover, we compared the utility of the BAT in two different populations: adults and children. The two populations studied were comparable in terms of sex ratio and allergy work-up performed.

We found differences between children and adults. First, only one child had severe symptoms, whereas 13 out of 21 adults (61.9%) did. According to the literature, the frequency of immediate severe reactions in children reportedly is lower than it is in adults [8]. We can speculate that in the paediatric population the mild-to-moderate reactions that occurred during a course of drug therapy were probably triggered by infections, even when they lasted less than one hour from drug intake. It has been suggested that most of the skin rashes that occur during beta-lactams treatments are due to the infection itself, with drug hypersensitivity being confirmed in less than 10% of cases [9]. To reinforce this hypothesis, 7 out of 18 children had never taken the drug before; so potential previous sensitization could be ruled out.

The second difference related to the average latency time between the culprit drug intake and clinical symptom appearance, which was 32 min (DS ± 24) for children and 13 min and 30 s (DS ± 17) for adults (*p* = 0.005). This difference is probably because even if the reaction occurred within one hour, cofactors played an important role in the drug reaction in children. An IgE-driven mechanism has been found more frequently in adults than in children.

The last difference observed between children and adults related to ST positivity. Among the children, only three patients had a positive ST result (16.6%), whereas thirteen adult patients (72.2%) had a positive ST result (*p* = 0.005).

In children, the OPT with the culprit drug is often required to reach a confident diagnosis in cases involving mild-to-moderate immediate drug reactions.

These two populations are not similar because of the differences in terms of the severity of the reactions, latency between last drug intake and symptom occurrence and ST results. These differences observed and discovered during the patients’ recruitment reflect the possible existence of different pathogenic mechanisms and triggers between children and adults.

In the adult population, when we compared the BAT results of patients with severe reactions (13 out of 21) and patients with mild reactions (8 out of 21), we found a BAT sensitivity of 38.4% and specificity of 100%. The BAT sensitivity obtained in this study is consistent with the sensitivity reported in the literature, which ranges from 28.6% to 55% for penicillin [10]. In addition, the specificity is aligned with the specificity reported in the literature, which is over 90% for penicillin (10).

Furthermore, 5 out of 13 patients (38.5%) with severe symptoms had a positive BAT result. Moreover, no adult patients with mild symptoms had a positive BAT result, suggesting that a grading symptom score is an index of the predictability of BAT positivity. The degree of similarity between symptom severity and BAT results was significantly different (*p* = 0.025) among adults.

Among children, the sensitivity and specificity could not be calculated because no children had a positive BAT result, and none of them had severe symptoms. According to our results, we can assume that the BAT does not increase the sensitivity of the allergy work-up because we did not find false negatives, which means patients with a negative ST or Immunocap-FEIA result and a positive BAT result. This result contradicts those presented in previous studies of adults (10) that showed that the combination of Immunocap-FEIA with BAT improves the sensitivity of the allergy work-up. The small number of patients probably affected our data.

In conclusion, in both paediatric and adult populations, BAT does not seem to be a useful tool to increase the sensitivity of an allergy work-up to diagnose immediate hypersensitivity to AMX or AMX-C.

In adults, we found a good correlation between BAT and ST results (61.9%) and a good degree of matching between symptom severity and BAT results (*p* = 0.025). These findings mean that the grading symptom score could be an index of predictability of BAT positivity. Therefore, BAT could likely be useful in the allergy work-up of adult patients with a history of severe symptoms to prevent them from undergoing ST that are not risk-free.
